# Altered excitatory and inhibitory neocortical circuitry leads to increased convulsive severity after pentylenetetrazol injection in an animal model of schizencephaly, but not of microgyria

**DOI:** 10.1002/epi4.12625

**Published:** 2022-07-21

**Authors:** Luiza dos Santos Heringer, Julia Rios Carvalho, Julia Teixeira Oliveira, Bruna Texeira Silva, Domethila Mariano de Souza Aguiar dos Santos, Anna Lecticia Martinez Martinez Toledo, Laura Maria Borges Savoldi, Debora Magalhães Portela, Suelen Adriani Marques, Paula Campello Costa Lopes, Ana Maria Blanco Martinez, Henrique Rocha Mendonça

**Affiliations:** ^1^ Neurodegeneration and Repair Lab, Department of Pathology, Postgraduate Program in Anatomical Pathology, Faculty of Medicine Universitary Hospital Clementino Fraga Filho, Federal University of Rio de Janeiro Rio de Janeiro Brazil; ^2^ D'Or Institute for Research and Teaching Rio de Janeiro Brazil; ^3^ Laboratory of Neuroplasticity, Department of Neurobiology, Institute of Biology Fluminense Federal University Niterói Brazil; ^4^ Integrated Lab of Morphology, Institute of Biodiversity and Sustainability NUPEM Multicentric Postgraduate Program in Physiological Sciences – SBFis, Federal University of Rio de Janeiro Brazil

**Keywords:** epilepsy, neuroplasticity, polymicrogyria, Schizencephaly, synaptogenesis

## Abstract

**Objective:**

Malformations of the polymicrogyria spectrum can be mimicked in rodents through neonatal transcranial focal cortical freeze lesions. The animals presenting the malformations present both altered synaptic events and epileptiform activity in the vicinity of the microgyrus, but the comprehension of their contribution to increased predisposition or severity of seizures require further studies.

**Methods:**

In order to investigate these issues, we induced both microgyria and schizencephaly in 57 mice and evaluated: their convulsive susceptibility and severity after pentyleneterazol (PTZ) treatment, the quantification of their symmetric and asymmetric synapses, the morphology of their dendritic arbors, and the content of modulators of synaptogenesis, such as SPARC, gephyrin and GAP‐43 within the adjacent visual cortex.

**Results:**

Our results have shown that only schizencephalic animals present increased convulsive severity. Nevertheless, both microgyric and schizencephalic cortices present increased synapse number and dendritic complexity of layer IV and layer V‐located neurons. Specifically, the microgyric cortex presented reduced inhibitory synapses, while the schizencephalic cortex presented increased excitatory synapses. This altered synapse number is correlated with decreased content of both the anti‐synaptogenic factor SPARC and the inhibitory postsynaptic organizer gephyrin in both malformed groups. Besides, GAP‐43 content and dendritic spines number are enhanced exclusively in schizencephalic cortices.

**Significance:**

In conclusion, our study supports the hypothesis that the sum of synaptic alterations drives to convulsive aggravation in animals with schizencephaly, but not microgyria after PTZ treatment. These findings reveal that different malformations of cortical development should trigger epilepsy via different mechanisms, requiring further studies for development of specific therapeutic interventions.

**Key points**

- Alterations of excitatory and inhibitory synapses within the paramicrogyral zone are not uniform among different malformations of the Polymicrogyria spectrum
- Schizencephaly, but not microgyria, promotes increased GAP‐43 content and increased dendritic spine density in the neocortex
- Schizencephaly, but not microgyria, is related to increased convulsive severity after pentileneterazol systemic injection

## 1 INTRODUCTION

It is estimated that around 40% of childhood epilepsy cases that are resistant to drug treatments are caused by developmental cortical malformations.^1^ Among these, malformations of the polymicrogyria (PMG) spectrum are the most common type of cortical malformations,^2^ stemming from both migrational and post‐migrational disturbances,^3^ due to genetic alterations or destructive lesions of the developing neocortex.^4^ PMG can be characterized by several histological patterns that arise from the abnormal development or loss of neurons in the cortical plate, which can happen simultaneously,^5^ leading to focal disruptions of the normal lamination pattern of the isocortex, as well as in the grooving process associated with the fusion of the molecular layer margin of the sulci, with or without leptomeningeal barrier between both sides.^5^, ^6^, ^7^, ^8^, ^9^ Within the spectrum of PMG, schizencephaly is a malformation characterized by clefts that extend through the cerebral cortex without fusion of adjacent molecular layers, bordered by polymicrogyric cortex.^10^, ^11^ Epilepsy is the most common disease caused by malformations of the PMG spectrum, being present in 78% of patients with PMG and 81% of patients with schizencephaly.^4^, ^12^, ^13^

Although the association of these malformations with high rates of intractable epilepsy is well established,^14^ the mechanisms by which these malformations contribute to the onset of epileptiform activity are poorly understood.^15^ Studies mimicking the physiopathology of PMG through neonatal freeze lesions (FL) in animal models demonstrated that there is a high probability to evoke epileptiform activity in the paramicrogyral zone (PMZ) upon stimulation.^16^, ^17^ However, there is a latency of 12 days after malformation induction for the onset of this epileptiform activity,^18^ suggesting that the plastic changes that occur during this period result in the onset of hyperexcitability.^19^ Besides the appearance of epileptiform activity in brain slices, freeze‐lesion induced‐microgyria leads to EEG recording of spike–wave discharges, particularly during sleep,^20^ and predisposes animals to longer‐lasting febrile seizures in PND10.^21^

The microgyri and the PMZ are characterized by an abnormal organization of afferent and efferent connections.^22^ A reorganization of the circuitry was demonstrated in the somatosensory cortex of rats, where a hyperinnervation of the thalamocortical circuits in the PMZ was present,^23^ as well as an increase in callosal afferents in this region, supporting the hypothesis of increased connectivity.^24^ Indeed, cortical slices from FL models present several changes in connectivity and excitability in the cortical network, such as the increase in the excitatory input both in interneurons and projection neurons located in the PMZ 14 days post‐lesion, where epileptiform activity was also demonstrated.^25^ Increased excitatory connectivity was also seen between cortical layers II/III and V through laser scanning photostimulation, which demonstrated an increase in the frequency of excitatory postsynaptic currents, characterizing a local hyperexcitable intracortical circuit.^26^

Despite synaptic function alterations have been described in animal models of microgyria, it is not known whether the hyperexcitability that causes epileptiform activity occurs due to an increase in the existing circuitry activation, or if it is correlated with the formation of morphological identifiable excitatory and/or inhibitory synapses. In addition, the study of epileptogenesis that occurs in schizencephaly has never been conducted in animal models. Thus, our study aims to evaluate the alteration of the excitatory and inhibitory synaptic circuitry in the FL model for the generation of microgyria and schizencephaly in mice, in order to elucidate the epileptogenic mechanisms of these malformations.

## 2 MATERIALS AND METHODS

Methods are described briefly within the article, and detailed methodological information is provided in the Methods S1.

C57bl/6 mice were employed in this study. Briefly, they were raised in a light/dark cycle (12 h:12 h), in a climate‐controlled environment throughout the trial period. All experimental procedures comply with the standards for the use of laboratory animals of the Scientific Experimentation of the Health Sciences Center of the Federal University of Rio de Janeiro registered within the National Council for the Control of Animal Experimentation (CONCEA) and the international standards for animal experimentation and were approved under protocol 018/20.

Fifty‐seven post‐natal day (PND) zero aged mice, of either sexes, were anesthetized and subjected to freeze‐lesion induced malformations of the PMG spectrum as described elsewhere.^22^, ^24^ At PND33, all animals were tested for convulsive susceptibility on the Modified Racine scale^27^ after systemic pentyleneterazol (PTZ) treatment (60 mg/kg, intraperitoneal). Animals that did not present morphologically identifiable malformations were discarded.

Immediately after the convulsive susceptibility test, animals were anesthetized and killed. Their dissected brains were freshly employed either for Western blot against the antibodies detailed in Table 1, ^28^; Golgi–Cox staining to analyze dendritic complexity and dendritic spines; or were fixed either for hematoxylin and eosin (HE) histology to classify the malformations; immunofluorescence against the antibodies detailed in Table 1; or transmission electron microscopy to analyze synapses.

**TABLE 1:** List of primary and secondary antibodies employed forimmunohistochemistry and western blot

	Source	Dilution	Manufacturer
Primary antibodies			
GAP‐43 (Ab75810)	Rabbit	1:100	Abcam
Gephyrin (sc25311)	Mouse	1:100	Santa Cruz
SPARC (AF942)	Mouse	1:100	R&D systems
Secondary antibodies			
Alexa 488 anti rabbit	Donkey	1:500	Abcam
Alexa 488 anti mouse	Donkey	1:500	Abcam
Western blotting			
Primary antibodies			
GAP‐43 (G9264)	Mouse	1:1000	Merck
Gephyrin (sc25311)	Mouse	1:500	Santa Cruz
Secondary antibodies			
Mouse anti‐igG		1:10 000	BioRad
Rabbit anti‐igG		1:10 000	BioRad

Graphs were plotted as means ± standard deviations and statistical analysis were performed using the GraphPad Prism 8.0 software. Shapiro–Wilk test was performed to confirm normal distribution. Statistical analyses were performed using one‐way ANOVA with Tukey's post‐test or Two‐way ANOVA followed by Bonferroni post‐test. Differences were considered significant when *p* ≤ .05.

## 3 RESULTS

### 3.1 Morphological characterization of the malformations

**FIGURE 1:**
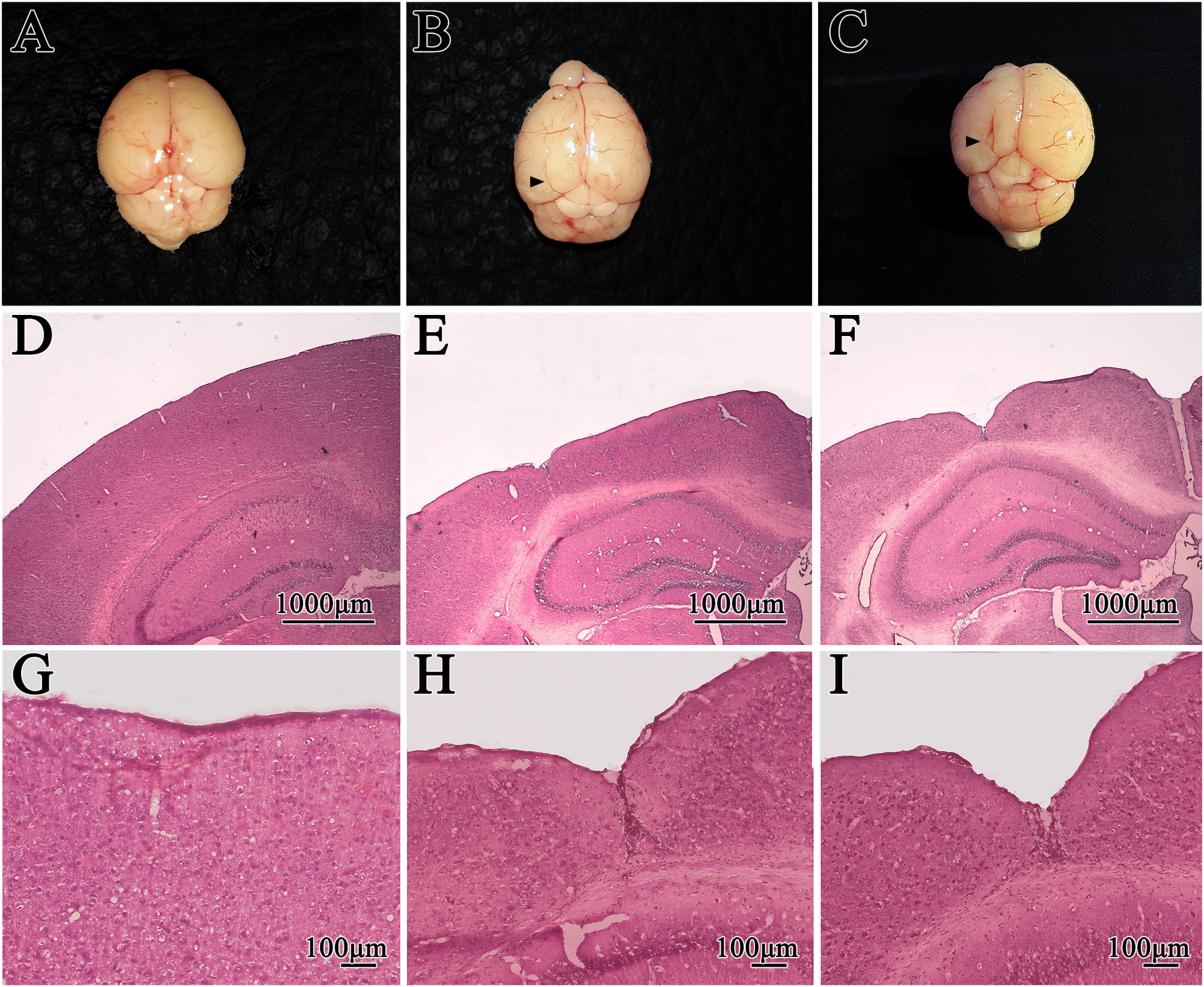
Macroscopic and histological characterization of cortical malformations. Macroscopic characterization of malformations: (A) Visual cortex of a control animal. (B) Visual cortex of a microgyric animal. (C) Visual cortex of a schizencephalic animal. (black arrows indicate malformations). Histological characterization of H&E stained malformations: (D,G) Histological sections of the visual cortex of a control animal, composed of six cortical layers. (E,H) Histological sections of the visual cortex of the a microgyric animal demonstrating the formation of a cortical microsulcus with fusion of the molecular layers, concomitant with a loss of the infragranular layers. (F,I) Histological sections of the visual cortex of a schizencephalic animal showing the loss of all cortical layers, with the formation of a cleft that extends to the hippocampus. (D,F 1000 μm scale bar; G,I 100 μm)

Cortical malformations were induced through neonatal transcranial focal freeze‐lesions, causing macroscopically recognizable microgyria and schizencephaly, in the animals subjected to lesions of 5 or 15 seconds of duration, respectively (Figure 1A‐C). By HE staining (Figure 1D‐I), we demonstrated that microgyria was characterized by the formation of a cortical microsulcus showing fusion of the molecular layer (Figure 1E,H), where discrete cell death occurred, with cortical tissue of apparently normal organization in the adjacent region, known as the PMZ. Alternatively, we demonstrated the formation of schizencephaly, characterized by greater loss of the cortical layers, forming a cleft lined by gray matter, which extends through all the isocortical layers to the hippocampus, between the non‐fused margins of molecular layers of the neighboring neocortices (Figures 1F,I).

### 3.2 Convulsive evaluation

**FIGURE 2:**
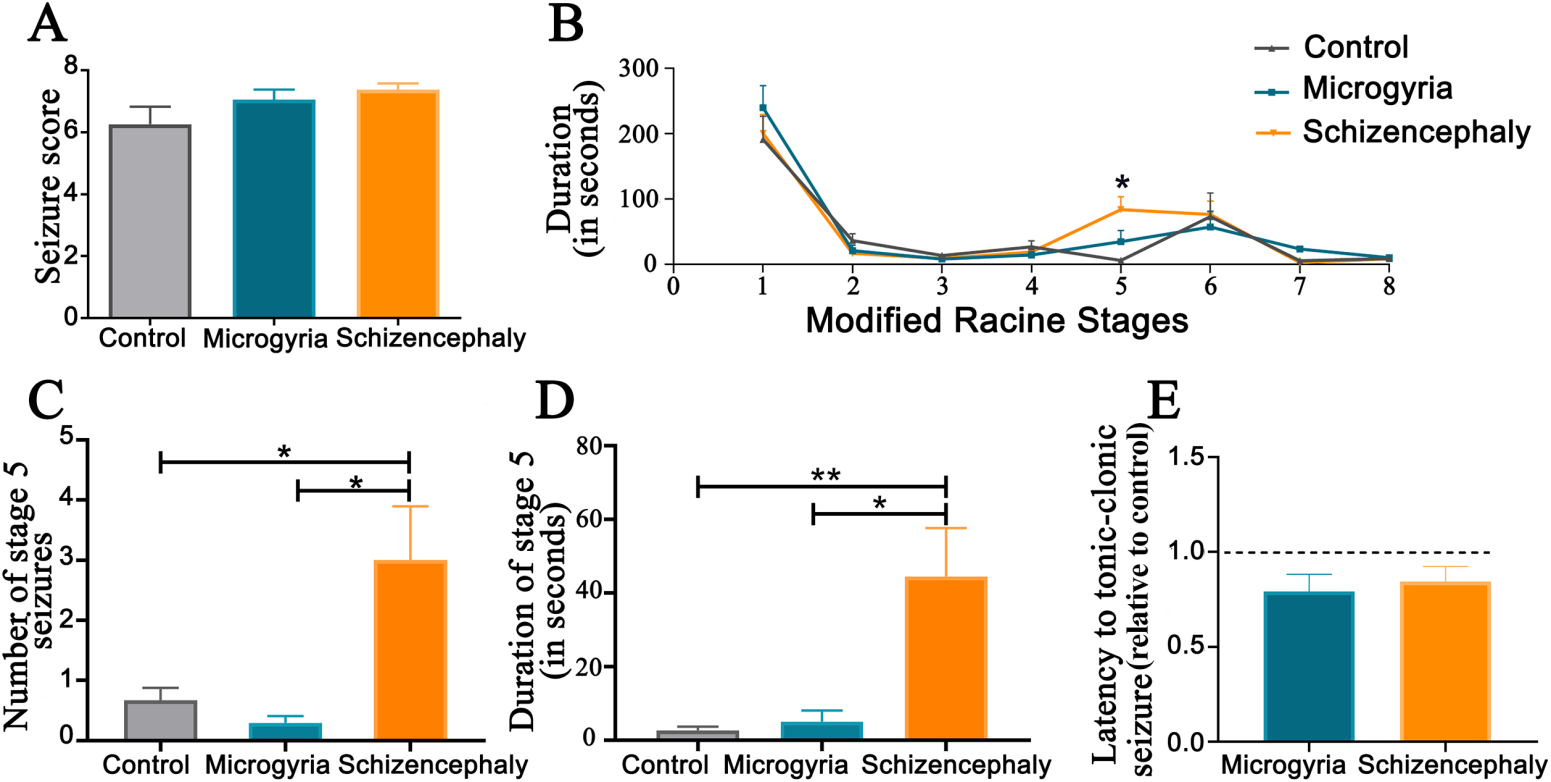
PTZ‐induced convulsion evaluation. (A) Score of seizure analysis. (B) Analysis of duration of the convulsive stages of the adapted Racine scale. (C) Analysis of the number of seizures in stage 5 of the adapted Racine scale. (D) Analysis of duration of seizures in stage 5 of the adapted Racine scale. (E) Analysis of latency for stage 8 of the adapted Racine scale. (N = 15: control, N = 18: microgyria, N = 24: schizencephaly) *p ≤ .05, **p ≤ .01. PTZ, pentyleneterazol

It is well known that freeze‐induced microgyria causes circuitry changes that lead to epileptiform activity in the PMZ.^19^ In order to verify if this local hyperexcitability is translated into increased seizure severity, we systemically applied the GABA A receptor antagonist PTZ, to induce convulsions. The assessment of seizures was performed using the adapted Racine behavioral scale, where we first analyzed the seizure score, where we did not find any difference between groups (Figure 2A). Then, we evaluated the duration in seconds that the animals remained in each of the modified Racine Scale stages. When comparing the groups, the Schizencephaly group showed increased duration of stay in stage number five (unilateral clonic seizures) when compared to the control group (Figure 2B). From this, we conducted a deeper analysis on stage number five separately. Since this stage is described by unilateral clonic movements, we evaluated the upperlimb myoclonic jerks contralateral to the lesion side, evaluating both the number and duration of the seizures. We found that the Schizencephaly group showed a significant increase in the count of contralateral clonic seizures on the injured side when compared to the Control and Microgyria groups (Figure 2C). Similarly, the analysis of seizure duration revealed that the Schizencephaly group presented a significant increase as compared to the Control and Microgyria groups (Figure 2D). The analyses of these parameters in the upperlimb ipsilateral to the lesion revealed no significant alterations among the groups studied (not shown).

### 3.3 Ultrastructural evaluation of synapses

**FIGURE 3:**
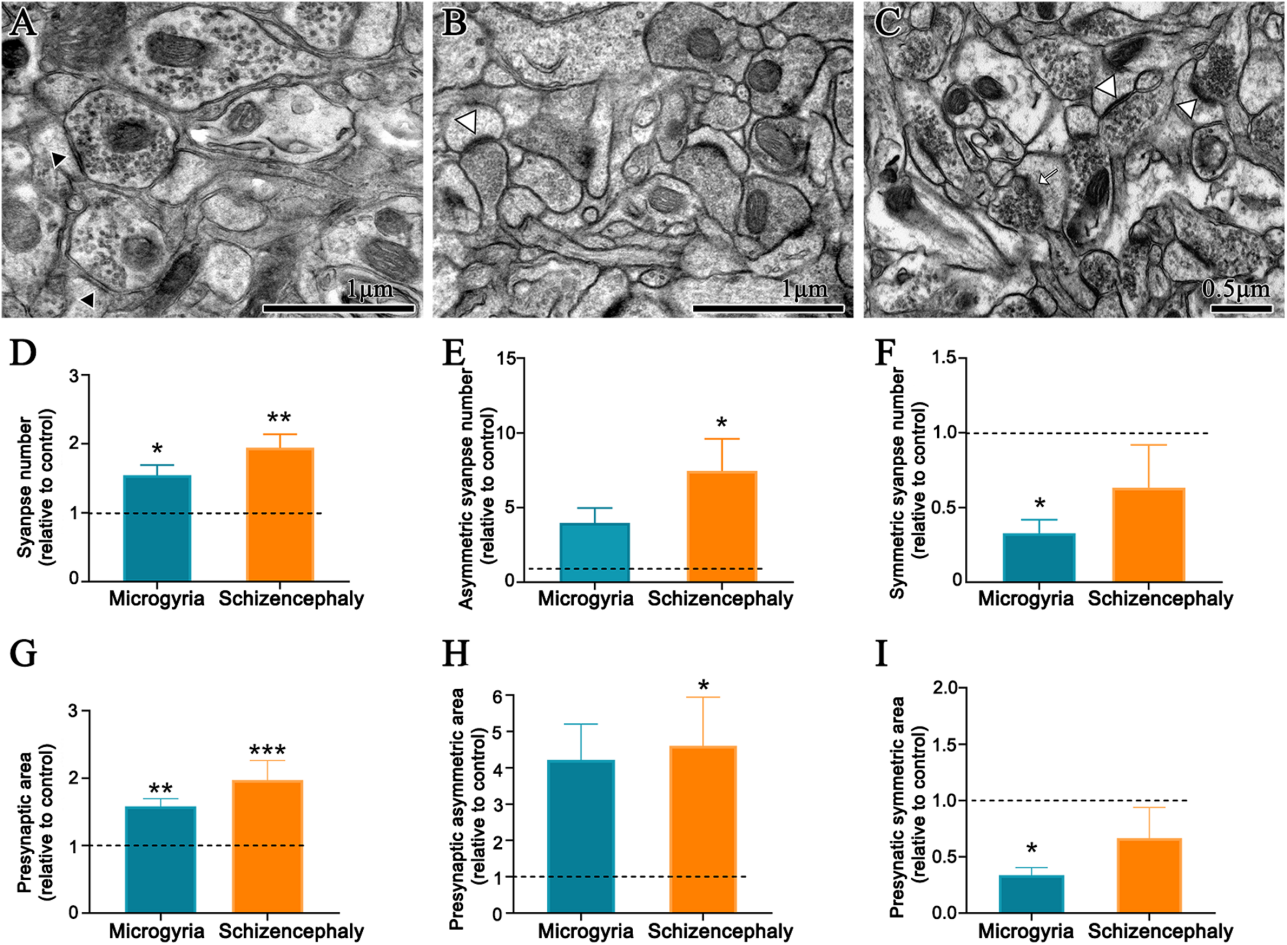
Malformed cortices present altered symmetric and asymmetric synapses. (A) Cortical neuropil of a control animal. (B) cortical neuropil of a microgyric animal. (C) Cortical neuropil from a schizencephalic animal (black arrowhead indicates symmetric synapses; white arrowhead indicates asymmetric synapses; arrows indicate non‐classifiable synapses). (D) Analysis of the number of synapses (n = 5: control, n = 4: microgyria and schizencephaly). (E) Analysis of the number of asymmetric synapses. (F) Analysis of the number of symmetrical synapses. (G) Analysis of the area of the presynaptic terminal of synapses. (H) Analysis of the area of the presynaptic terminal of asymmetric synapses. (I) Analysis of the presynaptic terminal area of symmetric synapses (n = 3: control, microgyria and schizencephaly). *p ≤ .05; **p ≤ .01

Altered number of excitatory and inhibitory synaptic events has been correlated to epileptiform activity generation in the PMZ. It has been shown in slices of the somatosensory cortex, a gradual increase of both spontaneous and miniature synaptic events that precedes epileptiform activity, resulting in increased excitatory Pos‐synaptic current (PSC)/inhibitory PSC ratio.^25^, ^29^ Although miniature PSC has been traditionally interpreted as alterations of synaptic number, it is known that presynaptic active zone size modulates the neurotransmitter vesicle release probability,^30^ making this assumption imprecise. Therefore, a definitive analysis of synapse number alterations requires ultrastructural studies. Thus, we have performed transmission electron microscopy analysis of the neuropil of layer V of the visual cortex (Figure 3A‐C). We have found that the cortex adjacent to both microgyria and schizencephaly presented an increased number of synapses, as well as the percent of the area occupied by the presynaptic terminals, when compared to control animals (Figure 3D,G). Additionally, asymmetric synapses number and their occupied area by the presynaptic terminals were increased in schizencephalic, but not in microgyric cortices (Figure 3E,H). Finally, symmetric synapses number and their occupied area by the presynaptic terminals were decreased in microgyric, but not in schizencephalic cortices (Figure 3F,I).

### 3.4 Dendritic analysis

**FIGURE 4:**
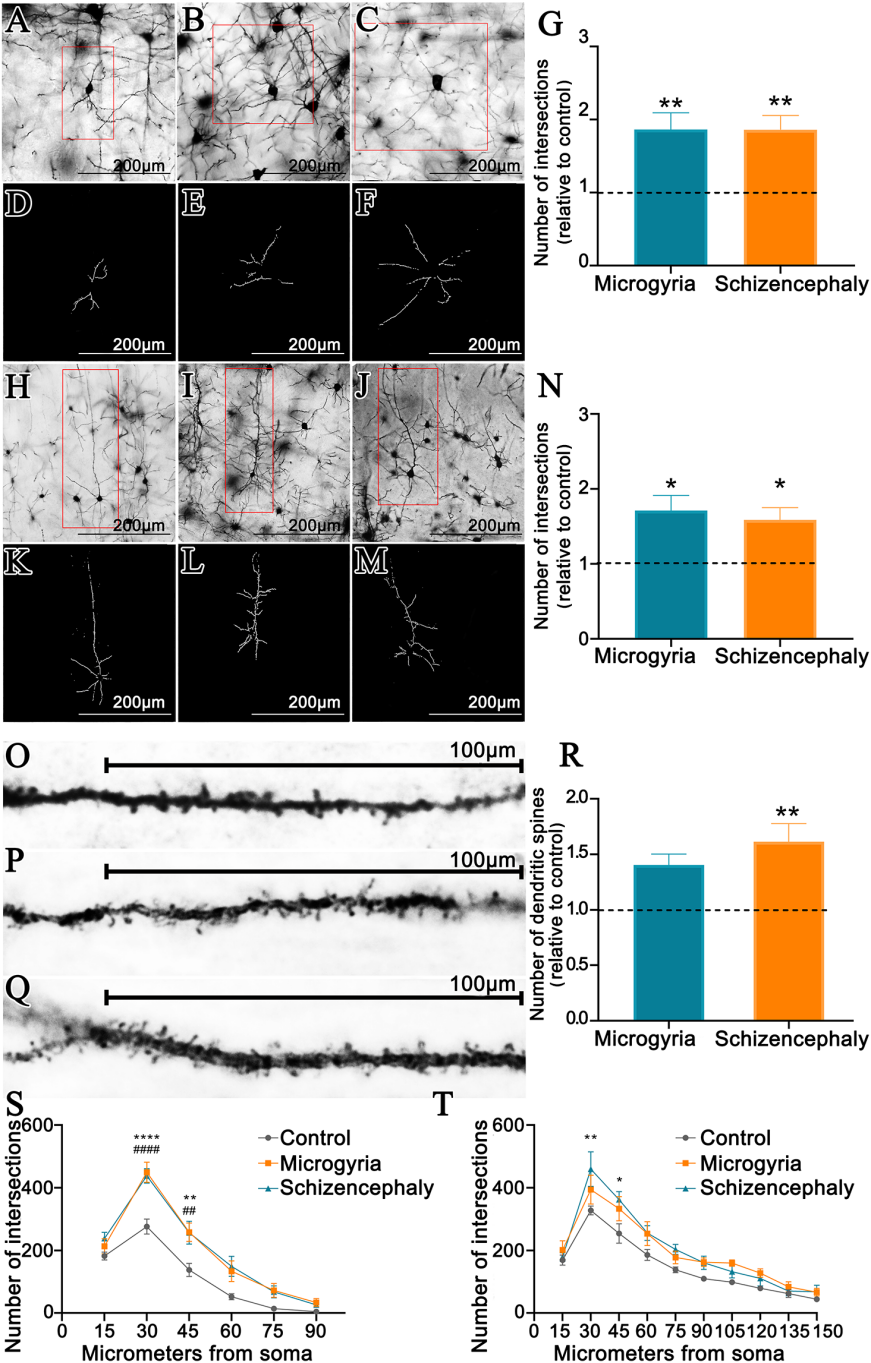
Dendritic morphological evaluation of layer IV‐located granular spiny neurons and layer V‐located pyramidal neurons. (A,C) Layer IV granular spiny neurons stained with Golgi–Cox. (D,F) Isolation of dendritic arborization of layer IV spiny granular neurons. (A,D) Control. (B,E) Microgyria. (C,F) Schizencephaly. (G) Dendritic arbor complexity of spiny granular neurons of layer IV. (H,J) Layer V pyramidal neurons stained with Golgi–Cox. (K,M) Isolation of dendritic arborization of layer 5 pyramidal neurons. (H,K) Control. (I,L) Microgyria. (J,M) Schizencephaly. (N) Dendritic arbor complexity of layer V pyramidal neurons. (O,Q) Dendritic spines from the apical dendrite of layer V pyramidal neurons stained with Golgi–Cox. (O) Control. (P) Microgyria. (Q) Schizencephaly. (R) Analysis of the number of dendritic spines. (S) Sholl analysis of layer IV spiny granular neurons. (T) Sholl analysis of layer V spiny pyramidal neurons.*Relative to schizencephaly; ^#^relative to microgyria. *p ≤ .05, **p ≤ .01; ^#^^#^ *p* ≤ .01; ^####^ *p* ≤ .0001 (n = 6: control, microgyria, and schizencephaly)

Since neocortical dendritic arbors can extend beyond the limits of the cortical layers, ultrastructural analyses of synapses do not identify which neuron is receiving the synapses. Golgi–Cox staining allows the study of the dendritic arbor complexity of morphologically identified neurons from different layers, with dendritic complexity being correlated to the number of synapses sites and the number of dendritic spines representing a subset of excitatory synapses.^31^ Thus, we investigated the dendritic complexity of granular spiny neurons located at layer IV (Figure 4A‐C) and of pyramidal neurons located at the layer V (Figure 4H‐J). Imaging of selected neurons of layer IV (Figure 4D‐F) and layer V (Figure 4K‐M) allowed the Sholl Analysis of the dendritic arbors. Our results revealed that the dendritic complexity was increased in both microgyric and schizencephalic groups either in layer IV‐located granular spiny neurons (Figure 4G) and layer V‐located pyramidal neurons (Figure 4N). Analysis of dendritic spines number in the apical dendrites of layer V‐located pyramidal neurons (Figure 4O‐Q) revealed an increased number of dendritic spines in the Schizencephaly group, but not Microgyria group (Figure 4R). Additionally, Sholl Analysis of layer IV‐granular neurons revealed that dendritic arbor complexity was increased until 45 μm from soma in both microgyric and schizencephalic cortices when compared to control group, while the same was found only in the schizencephalic cortex when layer V‐pyramidal neurons were analyzed (Figure 4S‐T).

### 3.5 SPARC content in the malformed cortices

**FIGURE 5:**
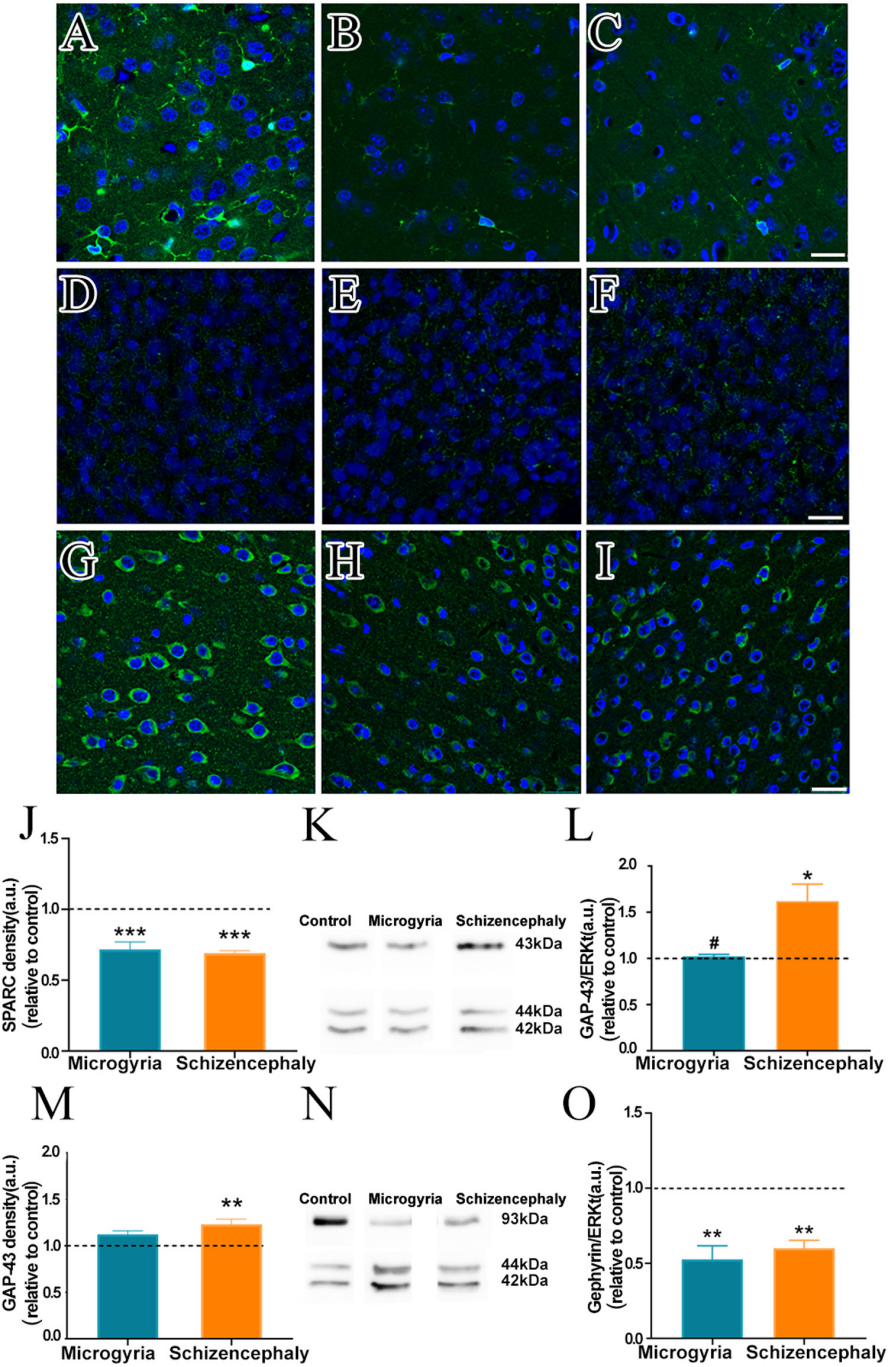
SPARC, GAP‐43 and gephyrin are altered in microgyria and schizencephaly. Immunofluorescence (green) for SPARC (A,C; n = 4 control, microgyria and schizencephaly), GAP‐43 (D,F; n = 3: control, microgyria and schizencephaly), gephyrin (G,I; (n = 3: control, microgyria and schizencephaly) and DAPI nuclear staining (blue) in the visual cortex of mice. (A,D,G) Control. (B,E,H) Microgyria. (C,F,I) Schizencephaly groups. Graphs of total section immunofluorescent mean gray value of SPARC (J) and (M) GAP‐43. Scale bar = 25 μm representative blots of the loading control, Erk, GAP‐43 (K; n = 3: control, microgyria and schizencephaly) and gephyrin (N; n = 3: control, microgyria and schizencephaly). Graphs and optic density analysis of Western blots against GAP‐43 (L) and gephyrin (O). *Relative to 1; ^#^relative to microgyria. *p ≤ .05; **p ≤ .01; ***p ≤ .001

The process of synapse formation is modulated by a plethora of membrane bound and soluble molecules that signals into neurons, instructing the formation of synaptic sites. Specifically, SPARC and SPARC‐like proteins are astrocyte‐derived factors that inhibit and promote neocortical synaptogenesis, respectively.^32^ We performed immunofluorescence against SPARC in the visual cortex adjacent to the malformations (Figure 5A‐C). Our results have shown that SPARC content was decreased both in microgyric and schizencephalic visual cortex when compared to the control group (Figure 5J).

### 3.6 GAP‐43 content analysis in the malformed cortices

Despite the overall increase in synapse number found in both malformed groups, asymmetric synapses were increased specifically in the Schizencephaly group. Since it has been recently shown that GAP‐43 upregulation induces excitatory but not inhibitory synaptogenesis in a rodent cortical dysplasia model,^33^ we performed immunofluorescence (Figure 5D‐F) and Western blot analyses of GAP‐43 content within the region adjacent to the malformations (Figure 5K). Our results have shown that schizencephalic, but not microgyric visual cortex, presents increased content of GAP‐43 when compared to control cortices (Figure 5L,M).

### 3.7 Gephyrin content in the malformed cortices

Despite the overall increase in synapse number found in both malformed groups, symmetric synapses were decreased only in the Microgyria group. In order to investigate the underlying reason for these differential synaptogenic mechanisms, we investigated the content levels of the postsynaptic scaffold protein of inhibitory neurons, gephyrin, by immunofluorescence (Figure 5G‐I) and Western blot (Figure 5N). Our results have shown that both microgyric and schizencephalic cortices present reduced levels of gephyrin in comparison to the control cortex (Figure 5O).

## 4 DISCUSSION

Animal models of microgyria have been widely used to investigate epileptogenic mechanisms within the PMZ during the last decades. From these studies, it was elucidated that PMZ receives enlarged thalamic and callosal projections, correlated to epileptiform activity recordings that can evolve to ictal activity when slices are perfused with magnesium‐free ACSF.^16^, ^24^, ^34^, ^35^ Increased excitatory spontaneous and miniature postsynaptic potentials can be recorded even before epileptogenic activity, suggesting that increased excitatory drive is required for epileptiform activity development.^29^ However, it is not currently known if the observed synaptic alterations results from altered activity of pre‐existing connections or if it is secondary to enhanced synaptogenesis. So, our study provides consistent evidence, showing that synapse number is increased both in microgyria and schizencephaly, with imbalances pending through excitatory synapses in both malformations. Principal neurons of layer IV and V present increased dendritic complexity, arguing in favor of increased synaptic sites within these neurons, which could contribute to hyperexcitability generation. Besides, only Schizencephaly group presents enhanced apical dendritic spine number. The hyperexcitability was confirmed for schizencephalic animals, which presented increased duration and number of episodes of unilateral myoclonic convulsive seizures after PTZ systemic injections. The synaptogenic processes underlying the epileptogenesis of the studied cortical malformations seems to rely on decreased SPARC and gephyrin contents in both malformed cortices, and increased GAP‐43 content in schizencephalic cortices. Thus, our study reveals for the first time that PMZ altered synaptic balance in schizencephaly leads to behaviorally assessed convulsions susceptibility, which was not found in microgyric cortex, despite the synaptic alterations found.

### 4.1 Neonatal focal cortical freeze lesion can elicit both microgyria and schizencephaly

The PMG spectrum malformations are likely to be the endpoint of different etiological processes during neocortical post‐migrational development, and can be identified by either magnetic resonance imaging or histopathology. Its causes can be either genetic—such as mutations of tubulins or collagen 4‐related genes, that regulates cortical organization—or disruptive—following neonatal cytomegalovirus infection and/or fetal cerebral ischemia, for example. Although the identification of its causes might be of prognostic value, the collection of symptoms of each patient also rely on the regions of the brain affected, its extent and other associated malformations. Thus, the comprehension of the outcome of PMG spectrum malformations described here, might be relevant to the management not only of malformations due to destructive origins, but also to genetic ones. Importantly, multi‐centric large cohort studies have found 20.3% of PMG cases to be of genetic causes, and when only patients presenting both PMG and epilepsies were included, the proportion of genetic origins dropped to 12%,^36^, ^37^ suggesting that most malformations of the PMG spectrum are due to destructive origins. Several works have demonstrated that a freezing transcranial focal lesion, when applied to rodents up to 48 hours after birth, results in microsulcus formation in the region of neuronal death, leading to focal disruption of the normal lamination pattern of the isocortex, as seen in the malformations of human PMG spectrum.^24^, ^35^, ^38^ Following the literature that has described that longer freezing times could induce cortical clefts similar to what is seen on human schizencephaly,^27^ the present study not only mimicked microgyria, but also schizencephaly, a more severe malformation of the PMG spectrum. The longer freezing time led to a pattern characterized by the loss of tissues stemming from the bulkier cortical layers, thus hindering the fusion of molecular layers and instead creating a cleft in the lesioned area. That cleft can either extend all the way to the hippocampus (open lipped) or present a thin and unorganized layer above the hippocampus (close lipped). Importantly, this is the first scientific work ever to explore epileptogenic mechanisms in an animal model of schizencephaly.

### 4.2 Animals with schizencephaly present increased convulsive severity after PTZ‐induced seizures

Although microgyria induced by neonatal freeze lesion promotes epileptiform activity in the PMZ,^39^ spike‐waves discharges and ictal activity during in vivo EEG recordings, behavioral accessed convulsions does not occur unless a second hit is performed, such as hyperthermia and PTZ systemic injections of infant or adult rats, respectively.^40^, ^41^ Our results show that microgyric animals do not present any alterations in the adapted Racine scale after PTZ systemic treatment, when compared to normal animals. The lack of convulsive effects of PTZ in microgyric animals found in our study was also found in the study performed by Kellinghaus et al, employing a different pharmacological approach.^41^ On the other hand, our schizencephalic animals presented increased duration and number of episodes of myoclonic unilateral seizures after PTZ systemic injection. The myoclonic seizures increase in schizencephalic group was only found in the upper limb contralateral to the malformation. The lack of difference in the excitability of the microgyric animals in our study, might reflect that the hyperexcitability evaluated by others through electrophysiological studies^16^, ^35^ will not always translate into altered convulsive behavior.

### 4.3 Malformed animals present altered synaptic connectivity

It has been shown that miniature and spontaneous postsynaptic currents are increased into individually recorded neurons located at the PMZ.^29^ Since these electrophysiological evaluation does not resolve if the increased activity relies on increased activity of the pre‐existing circuitry or if it involves novel synaptogenesis, we have quantified synapses by transmission electron microscopy and investigated dendritic morphology by Golgi–Cox staining. We have found increased synapse numbers both in microgyria and schizencephaly. These synapses may target either layer IV‐located granular spiny neurons, and layer V‐located pyramidal neurons, that presents increased dendritic complexity. Layer IV‐located granular spiny neurons are the main cells that receive thalamic‐derived sensory inputs. Since sensory stimulation is an important factor precipitating seizures in patients with cortical malformations,^42^ the increased dendritic complexity of these neurons might predispose the animal to sensory‐induced seizures. In addition, increased complexity of layer V‐located pyramidal neurons might facilitate the activation of subcortical targets, such as inferior motor neurons located at the brainstem and spinal cord, that ultimately would lead to the behavioral convulsions. Interestingly, only schizencephalic animals presented an increased number of dendritic spines of the layer V‐located pyramidal neuron apical dendrites. Since initiation of burst activity during electrographic seizures induced by bicuculline occurs at the apical dendrites of layer V‐located pyramidal neurons, later spreading to the other components of its dendrites and soma,^43^ it is plausible to suppose that the increased number of excitatory synapses in the apical dendrites is one of the reasons why schizencephalic animals undergoes increased PTZ‐induced convulsive severity, while microgyric animals do not. Indeed, the decreased dendritic spines found on pyramidal neurons of Down Syndrome post‐mortem infants neocortex is speculated to be one reason for their lack of predisposition to epilepsy.^44^

Interestingly, our ultrastructural analyses revealed that excitatory synapses are specifically increased in the schizencephalic brain, whereas inhibitory synapses are specifically decreased in the microgyric cortex. The decreased inhibitory synapses found in the microgyric cortex might be a result of the loss of parvalbumin‐positive interneurons within the microgyral neocortex within the PMZ.^45^ On the other hand, Jacobs and Prince described non altered spontaneous inhibitory PSC into pyramidal neurons of the PMZ.^25^ This discrepancy may be explained by the increase in frequency discharge of low‐threshold spiking interneurons activity and the decrease in firing rate of fast‐spiking interneurons, which are also reduced in number in the PMZ.^45^, ^46^ Alternatively, post‐synaptic currents' kinetics depend on the molecular composition of the different subtypes of neurotransmitter receptors and transporters. Kamada et al have found increased GluN1, GluN2a, and GluN2b subunits of NMDA receptors and GLT1 and GLAST in the PMZ of adult animals.^47^ Conversely, Redeker et al have shown that neonatal freeze‐lesion leads to widespread reduction in GABA A receptor subunits α1, α2, α3, α5, and γ2 not only in the PMZ, but also in the contralateral cortex, in the adulthood.^48^ Thus, further understanding of molecular components associated with the kinetics of synaptic currents might provide the identification of molecular targets for translational studies.

### 4.4 Possible mechanisms of synaptogenic modulation in microgyria and schizencephaly

Our findings suggest possible mechanisms for the synaptogenic modulation after cortical malformations. Firstly, gephyrin reduction in the cortices of both malformations imply a decreased maturation of inhibitory circuitry.^49^ Maturation of inhibition is a master regulator of critical period closure within the visual cortex.^50^ Without closure of the critical period, axon remodeling and synaptogenesis are expected to occur in response to internal or external stimuli. In line with this idea, we have found SPARC reduction in both groups. The lack of this anti‐synaptogenic factor may allow synapse formation in malformed cortices. Indeed, SPARC has been shown to inhibit synaptogenesis in the visual cortex.^27^ Additionally, we have found increased GAP‐43 content in the schizencephalic cortex. Besides being a protein involved in axon elongation and remodeling,^51^ it has recently been shown that GAP‐43 stimulates the increase of excitatory, but not inhibitory postsynaptic density scaffold proteins, contributing to epileptogenesis of a cortical dysplasia animal model.^33^

## 5 CONCLUSION

Our study provided a detailed morphological description of the similar but not identical connectivity alterations that occur in the PMZ of microgyric and schizencephalic neocortices, suggesting possible mechanisms that might instruct them. The comparative analysis of microgyria and schizencephaly provided by the present Manuscript is important to differentiate how distinct malformations promote seizures with different severities, identifying novel and specific cellular and molecular targets for therapeutic interventions. Finally, our findings debut the investigations of the epileptogenic mechanisms of schizencephaly in animal models. Thus, we hope to encourage other scientists to study different aspects of schizencephaly, providing hope for the patients that present this malformation around the globe.

## CONFLICT OF INTEREST

The authors declare that they do not have any competing interests. We confirm that we have read the Journal's position on issues involved in ethical publication and affirm that this report is consistent with these guidelines.

## Supporting information


Methods S1
