# Supplementary material for: Altered excitatory and inhibitory neocortical circuitry leads to increased convulsive severity after pentylenetetrazol injection in an animal model of schizencephaly, but not of microgyria
Source: Epilepsia Open. 2022 Jul 21;7(3):462–73. doi: 10.1002/epi4.12625 (PMC9436300; doi:10.1002/epi4.12625)
Supplement: Supplementary file 1 — Methods S1 [file EPI4-7-462-s001.docx]

**Supplementary Methods**

**2.1 Animals**

C57bl/6 mice were raised in a light/dark cycle (12h:12h), in a climate-controlled environment throughout the trial period. All experimental procedures comply with the standards for the use of laboratory animals of the Scientific Experimentation of the Health Sciences Center of the Federal University of Rio de Janeiro registered within the National Council for the Control of Animal Experimentation (CONCEA) and the international standards for animal experimentation and were approved under protocol 018/20.

**2.2 Induction of cortical malformation**

Mice  were deeply anesthetized by hypothermia and had the skull exposed by incision with a number 10 scalpel blade, on post-natal day (PND) 0. A copper probe with tip measuring 5 x 2 mm, cooled to -55ºC with liquid nitrogen, was placed on the surface of cranial bone 2 mm left from the midline immediately anterior to the lambda for 5 seconds to induce microgyria and for 15 seconds to induce schizencephaly in the developing primary visual cortex. The animals had their skin sutured, and were placed under incandescent light to recover from anesthesia, when the animals were returned to their home cages. Control group animals were subjected to every procedure performed to induce PMG spectrum malformation, but with the copper probe at room temperature.

**2.3 Convulsions susceptibility test**

In PND33, the animals received an intraperitoneal injection of pentylenetetrazol at a convulsive dose (60mg/kg) dissolved in dimethylsulfoxide. They were then evaluated for duration in each behavioral phase of the adapted Racine scale over a period of 0-10 minutes after drug administration and/or initial latency until the appearance of a tonic-clonic seizure. The adapted Racine scale is divided into eight phases: 1: imprisonment of the gaze; 2: orofacial spasms; 3: neck snatch; 4: bilateral clonic seizure in sitting position; 5: unilateral clonic seizures; 6: tonic seizure with postural maintenance; 7: tonic seizure with postural loss; 8: tonic-clonic seizure. In addition, we evaluated the Seizure Score, where we scored the maximum phase that each animal reached on the Racine scale^22^.

**2.4 Tissue fixation for histology and immunohistochemistry**

In PND33, the animals were deeply anesthetized and intracardially perfused with a 4% paraformaldehyde fixative solution in 0.1M phosphate buffer pH 7.4. The animals were dissected, the brains collected, searched for malformations and post-fixed in 4% paraformaldehyde for 24 hours. Then the  brains were cryoprotected in sucrose solutions at concentrations of 10%, 20% and 30% (twenty-four hours each) in 0.1 M phosphate buffer (pH 7.4).

Subsequently, the brains were frozen by immersion in dry ice  for twenty minutes, and, in the region of the visual cortex, 14 µm coronal histological sections were obtained in the cryostat (Leica CM 1850). All sections were collected on previously gelatinized slides and stored for immunohistochemistry or Hematoxylin and Eosin staining.

**2.5 Hematoxylin and Eosin staining**

For Hematoxylin and Eosin (HE) staining, we used frozen coronal histological sections. Slides were rehydrated for 1 minute in distilled water, then stained with Mayer's hematoxylin for 3 minutes, followed by a rinse in tap water for 5 minutes. After washing, slides were stained with eosin for 1 minute, and then dehydrated in increasing alcohol baths (70%, 90%, 100%) for 1 minute each. At the end of dehydration, they were mounted with entellan, and taken to a brightfield microscope (Olympus BX51) to be analyzed for malformation characterization. The malformation was classified as Microgyria when the pia-mater of adjacent molecular layers were fused within the microsulcus. The malformation was classified as Schizencephaly when the pia mater of adjacent molecular layers were not fused, bordering a cortical cleft.

**2.6 Immunohistochemistry**

The slides were washed in phosphate buffered saline (PBS) pH 7.4 for 5 minutes. For tissue antigen retrieval, the slides were incubated with 1% sodium dodecyl sulfate (SDS) solution for 5 minutes, then washed twice in PBS. To block binding to non-specific sites, a solution of PBS containing 0.3% triton X-100 and 2.5% normal donkey serum (NDS) was used for 1 hour at room temperature. Then, except for negative controls, tissues were incubated with primary antibodies (Table 1) diluted in the blocking solution for twenty-four hours at room temperature.

Slides were washed in PBS, 3 times for 5 minutes each and then incubated with secondary antibodies (Table 1) diluted in the blocking solution for two hours at room temperature. Then, they were washed in PBS 4 times for 5 minutes each, and incubated for 5 minutes with the nuclear marker 4,6-diamino-2-phenylindole (DAPI, 20mg/mL, Sigma). At the end, the slides were washed in PBS once for 5 minutes, and mounted with fluoromount  (Electron Microscopy Science), and then stored in the freezer at -20ºC for further analysis. The absence of primary antibody was used as a negative control for the reaction. Labeling density was calculated using images captured on a fluorescence microscope (Olympus BX51), with a 40x objective. Four photomicrographs of the primary visual cortex distancing at least 42 micrometers rostrocaudally from each other and 0.5mm lateral and medial to the presumed lesion site were performed per animal. The captured images were quantified using ImageJ2 software. The “Mean gray value” parameter was evaluated to each image, from which the optical density was quantified from the average value of each photomicrograph. The results were presented as fold-changes when compared to controls, which were normalized to 1.

**2.7 Golgi-Cox Staining**

 The animals were deeply anesthetized and then decapitated. Their freshly removed brains were rinsed in water and placed in a Golgi-Cox solution (20.8% volume of 5% potassium dichromate solution; 20.8% volume of 5% mercury chloride solution, 16.6% volume of 5% potassium chromate solution, 41,8% volume in distilled water) for 15 days at 37ºC in the dark. Two hundred µm sections were acquired using a vibratome (Vibratome Series 1000, Sectioning System) in 6% sucrose solution medium, and the sections were processed using the free floating technique.

Protected from light, the sections were washed in distilled water once for 5 minutes, incubated in ammonium hydroxide 28% for 30 minutes, followed by washing in distilled water for 5 minutes, and fixed in a Kodak fixative solution for 30 minutes. The sections were then washed in distilled water once for 5 minutes, dehydrated in increasing concentrations of ethanol for 1 minute each (50%, 70%, 80%, 90% and 100%), and clarified in xylene for 3 minutes in 3 sequential baths. The sections were transferred to gelatinized slides and mounted with Entellan. For further analysis of the complexity of dendritic arborization, two images of the visual cortex distancing at least 200 µm rostrocaudally from each other were captured in a brightfield microscope (Olympus BX51) with a 20x objective, containing granular spiny neurons from layer IV, and pyramidal neurons from layer V in the regions distancing 0.5 millimeters lateral to the presumed lesion site. Three neurons were focused in each photomicrograph, providing the analysis of 12 neurons per animal (6 and 6 pyramidal neurons). The images were processed using the ImageJ2 software, using the “Sholl Analysis” plug-in.

Briefly, in the two-dimensional images of each neuron, the photomicrographs were transformed into black and white images. Neurites belonging to non-selected neurons were manually erased. From this, a straight line was drawn with the “straight” tool where the beginning of the tracing would originally be the location of the nuclei, up to the farthest point of the dendritic arbor. From this tracing, Sholl analysis was performed using the “Sholl Analysis” plug-in, where concentric circles were considered with increments of 0,17 µm from the beginning to the end of the marked line. The evaluation of dendritic arborization of neurons was analyzed in terms of the sum of intersections of neurites with all circles, from the soma until the end of the dendritic arbor. Additionally, the sum of intersections within every 15 µm were compared among the groups studied.

For analysis of the number of dendritic spines, images were obtained in a brightfield microscope (Olympus BX51) at a 40x magnification, with further 2x optical amplification. Apical dendrites in layer I/II of pyramidal neurons located in layer V were quantified, with a total of 3 neurons per animal. The area of dendritic spines accounted for in each neuron was 30µm, leading to the analysis of 90µm of apical dendrites per animal.

**2.8 Transmission electron microscopy (TEM)**

For the ultrastructural analysis, the animals were submitted to intracardiac perfusion with 4% paraformaldehyde, 2.5% glutaraldehyde (GA) diluted in 0.1M phosphate buffer in DPN33. The brains were sectioned coronally with a thickness of 500µm in the vibratome (Vibratome Series 1000, Sectioning System). The slices containing the visual cortex were microdissected with the aid of a stereoscope (Nikon SMZ645), collecting a fragment measuring 1 mm width. This fragment containing the primary visual cortex of the area distancing 0.5mm lateral to the lesion in the injured groups, or the presumptive site of the cortical lesion in the control group, were placed in fresh fixative solution (2.5% GA in 0.1 M phosphate buffer) for 24 hours, and then processed to routine TEM. Processing for ultrathin sections consists of steps divided into five days:

1^st^ day: after fixation by immersion in the aforementioned solutions, the samples underwent two sequential five-minute washes in 0.1 M phosphate buffer (pH 7.4), followed by two five-minute washes in 0.1 M cacodylate buffer ( pH 7.4) and post-fixed for 2 hours in 1% osmium tetroxide containing 0.8% potassium ferrocyanide in 0.1 M cacodylate buffer (pH 7.4). The sections were washed 3 times for 5 minutes in 0.1 M cacodylate buffer (pH 7.4) and then twice for 5 minutes in distilled water. Then they were placed in a 1% uranyl acetate solution and left in this solution overnight.

2^nd^, 3^rd^ and 4^th^ day: the following day, samples were dehydrated in a graded acetone battery (30%, 50%, 70%, 80%, 90% - 2 washes for 10 minutes at each concentration) and 100% 2 washes for 20 minutes). Then, they were infiltrated in Poly/Bed 812 resin (Polysciences) and acetone in proportions (1:3, 2:2 and 1:1) for one night each, and in 100% resin for 24h.

5^th^ day: the samples were embedded in resine and kept at 60ºC for 48 hours for polymerization.

Later days: After the polymerization, ultra-thin coronal sections (70nm) were obtained in an ultramicrotome (PowerTome X-RMC), and collected on copper grids. These grids were taken to the transmission electron microscope (JEM1011 Jeol) for ultrastructural analysis. Images were acquired in the TEM JEM1011 (Jeol), with a magnification of 30,000X in a systematic way, alternating between the upper or lower corners of the mesh in the grid, in neuropil regions at a depth of 75% of the cortical plate, corresponding to layer V of the visual cortex. Fifty micrographs were taken per grid of each animal, leading to 250 photomicrographs per group. Image J software was used to quantify the total number of synapses, the relationship between excitatory and inhibitory synapses, and the area of the presynaptic terminal. The total number of synapses was quantified in the neuropil of the visual cortex, and described as the total number of synapses per analyzed area. A total area of 7,890µm² was accounted for in each electron micrograph, accounting for a total area of 394,500µm² per animal. For this quantification, synapses with synaptic vesicles in the presynaptic element and visible presynaptic and postsynaptic membrane specializations with or without evident synaptic cleft were considered. The results were presented as fold-changes when compared to controls, which were normalized to 1. To analyze the area of the presynaptic terminal, using the Image J software, the scale bar was set at 1 µm², and we used the “Measure” plug-in in the terminals marked with the aid of “Polygonal Selections” to obtain the area. All presynaptic terminals of previously quantified synapses were measured. The results were presented as fold-changes when compared to controls, which were normalized to 1.

**2.9 Western Blotting**

At DPN33, the animals were deeply anesthetized and then decapitated. The brains were freshly removed, dissected, and  1 mm segments of the visual cortex were removed in the area 0.5mm lateral to the lesion in the injured groups, and in the presumptive location of the cortical lesion in the control group. The dissected tissues were transferred to a homogenizer and macerated in a solution of 100µL of homogenization buffer, containing 10% Thermo Scientific RIPA buffer (25mM Tris•HCl pH 7.6, 150mM NaCl, 1% NP-40, 1% sodium deoxycholate, 0.1% SDS), 1% of the protease and phosphatase inhibitor cocktail (Thermo Scientific - 100mM AEBSF-HCl + 80μL aprotinin + 5mM betahistine + 1.5mM E-64 + 0.5M EDTA + 2mM leupeptin + 1mM pepstatin A), 1% EDTA (Thermo Scientific) and 88% MilliQ H2O. The entire process was carried out in ice.Protein dosage was performed following sample preparation using the Bradford method^23^. Once the amount of protein was known, we adjusted the samples using 4x sodium dodecyl sulfate (SDS) and the homogenization buffer, so that all samples had a standardized protein concentration (40μg/μL), being then stored in the freezer at -20°C until they were submitted to electrophoresis.Before starting the electrophoresis, 10 μL of the samples and 5μL of the Kaleidoscope Prestained Broad Range SDS-PAGE Standards (BioRad) color standard were applied on a 10% SDS/PAGE gel. Afterwards, the proteins from the samples were separated by molecular weight through electrophoresis with continuous running for approximately 90 minutes at a fixed amperage of 0.03A. Then, the proteins present in the running gel were transferred to a PVDF membrane (Amersham Biosciences) by electrophoresis for 1 hour with a fixed voltage of 10V.The transfer efficiency was observed by revealing the proteins in the membrane, with Ponceau's red staining.Subsequently, to detect the proteins, the membrane was treated with methanol and milliQ H_2_O for 1 minute each, and then incubated in a solution of Tris-buffered saline with 0,1% of Tween 20 (TBS-T). TBS-T + 5% defat milk was employed to block nonspecific membrane sites for 2 hours at room temperature. After blocking, membranes were incubated overnight with the primary antibodies (Table 1) diluted in block solution. The next day, the membrane was washed with TBS-T for 3 times of 10 minutes and incubated for 1 hour and 30 minutes with the specific secondary antibodies (Table 1) diluted in block solution. After this period, the membranes were washed again in 3 steps of 10 minutes in the TBS-T and then were developed in the Chemi-Express device (Loccus) after exposure to the ECL reagent (Amersham-Biosciences).

In order to prove that all lanes were loaded with the same amount of protein, the membrane stripping protocol was carried out with a 0.2M glycine solution pH 2.2, for 30 minutes under agitation, which leads to the removal of antibodies attached to the membrane. Then, we performed the same protocol for blocking non-specific sites, incubation with antibody against α-tubulin protein, used as loading control, secondary antibody and ECL reagent for band detection.After development, the films were digitized using the Adobe Photoshop CS3 program and optical density analysis of the bands was performed using the Scion Image software. The results of optical density analysis were divided by the loading control optic density to correct for eventual loading discrepancies, and the results were presented as fold-changes when compared to controls, which were normalized to 1.

**2.10 Statistical Analysis**

Graphs were plotted as means + standard deviations and statistical analysis were performed using the GraphPad Prism 8.0 software. Shapiro-Wilk test was performed to confirm normal distribution. Statistical analyzes were performed using one-way ANOVA followed by Tukey's post-test or Two-way ANOVA followed by Bonferroni post-test. Differences were considered significant when p ≤ 0.05.
